# Dental Undergraduate Students’ Perceptions of Blended Learning in the COVID-19 and Post–COVID-19 Years: Survey Study

**DOI:** 10.2196/63453

**Published:** 2025-11-28

**Authors:** Xin Li, Li Peng, Heng Tu, Lijia Liu, Xianglong Han, Ling Ye, Jing Yang

**Affiliations:** 1 State Key Laboratory of Oral Diseases, National Clinical Research Center for Oral Diseases, West China Hospital of Stomatology, Sichuan University Chengdu, Sichuan China

**Keywords:** blended learning, undergraduate dental education, student satisfaction, dental student, Chinese university, COVID-19

## Abstract

**Background:**

The outbreak of the COVID-19 pandemic created significant challenges but also a unique opportunity, accelerating the evolution of higher education, including dental education. This encouraged dental education to adopt more flexible modes like blended learning.

**Objective:**

This study aimed to explore senior undergraduate dental students’ views on blended learning during and after the COVID-19 pandemic and to identify modifiable factors influencing their engagement.

**Methods:**

A survey was conducted among final-year undergraduate students at a top-ranking dental school in mainland China during the fall semesters of 2020-2021 and 2023-2024. The survey assessed satisfaction with blended learning, preferences for engagement, strengths compared to purely online or offline teaching, and factors influencing engagement during and after the pandemic.

**Results:**

Response rates were 75% (85/114) in 2020 and 73% (47/64) in 2023. Blended learning was used in 53% (26/49) of evaluated courses. High satisfaction was reported by 82% (93/114) in 2020 and 59% (38/64) in 2023, with significant differences between high- and low-satisfaction groups (*P*<.001). Satisfaction with specific course types and learning activities was analyzed. Factors associated with higher satisfaction were evaluated using Pearson correlation. Students acknowledged the strengths of blended learning over online- or offline-only formats. In total, 70% (80/114) in 2020 and 61% (39/64) in 2023 expressed a desire to participate in blended dental education. Factors decreasing engagement included unstable technical support (68/114, 60% in 2020 vs 26/64, 41% in 2023), poor online-offline integration (58/114, 51% vs 34/64, 53%), lack of motivation (51/114, 45% vs 24/64, 38%), and insufficient teacher-student interaction (44/114, 39% vs 20/64, 31%). Factors increasing engagement included high-quality learning materials (76/114, 67% vs 43/64, 67%) and improved technical environments (62/114, 54% vs 35/64, 55%).

**Conclusions:**

Final-year dental students were generally satisfied with blended learning and recognized its strengths compared to purely online or offline formats, both during and after the pandemic. More efforts are required to enhance students’ potential engagement in blended learning for the future.

## Introduction

Undergraduate dental education aims to provide dental students with basic dental sciences and high-quality clinical practice to guarantee their competency [1,2]. Learning modes in dental education in China have continuously developed over the past decades. Generally, undergraduate dental courses are concentrated in the last 2 years, especially the final year, which mainly focuses on intensive and comprehensive training to develop students’ competence in clinical practice [2,3]. Traditional undergraduate dental education relies on textbook-based learning, focusing on classic textbooks in a specialized field for knowledge acquisition, but some of this information may be outdated, and students may develop fixed ways of thinking [2]. In face-to-face learning, students receive lectures from teachers in the classroom and usually acquire knowledge passively [4]. To alter the teacher-centered mode, problem-based learning and case-based learning questionnaires have been introduced, which support a learner-centered mode to foster active learning and clinical decision-making [5-7]. In recent years, advances in internet infrastructure have broadened the availability of internet services and prompted the application of online learning in modern dental education, exhibiting comparable or even better effectiveness compared to traditional face-to-face learning in dental education [8,9].

Blended learning, a learning mode that has grown increasingly popular in higher education since the 1990s [10-13], is generally defined as the integration of face-to-face learning with online learning [14]. This learning mode is designed to fully harness the merits of online learning and offline learning, enhance the effectiveness and delivery of educational systems, and facilitate learning activation and support [12,15]. While heterogeneity among different surveys exists, influencing factors of 3 dimensions, namely, learning dimensions, teaching dimensions, and curriculum dimensions, have been identified as accounting for student satisfaction with blended learning experiences in higher education in China [16]. Hitherto, only a few panel studies have focused on students’ opinions about the implementation of blended learning in dental education compared with face-to-face learning or online learning modes. For example, in early preclinical dental education for prosthetic dentistry, students highly evaluated blended learning with high satisfaction for learning content and outcomes, and appreciated blended learning for the achievement of learning objectives with the aid of e-learning tools [17]. In third-year undergraduate dental education for orthodontics, blended learning combining the use of plaster models with digital 3D models received positive feedback from students, and the learning performance outcomes were highly evaluated [18]. In final-year undergraduate dental education for oral radiology, dental students appreciated the benefits of blended learning in effectiveness, motivation, and engagement compared to the face-to-face only mode [19]. These findings are encouraging regarding the value of blended learning in undergraduate dental curricula.

The outbreak of COVID-19, an infectious disease caused by SARS-CoV-2, started in China in December 2019, was declared by the World Health Organization as a public health emergency of international concern in January 2020, and was assessed as a pandemic in March 2020 [20,21]. The immediate impacts of the COVID-19 pandemic on medical education are largely attributed to the modification of the educational calendar, online shifts of preclinical theoretical curricula, limitations on clinical rotations, and reliance on e-learning technology for clinical practical training [7]. By contrast, students were faced with unexpected challenges related to the COVID-19 pandemic [22,23]. Studying the learning mode differences between the COVID-19 pandemic and the post–COVID-19 years might provide valuable information for medical education. The competency of health professions students is also important [24], although it was not included in this study.

Indeed, interpreting the effects of the COVID-19 pandemic and postpandemic period on the learning outcomes and competency acquisition of students could provide clues for future dental education [25,26]. Existing studies on dental students’ self-evaluation of blended learning during the COVID-19 pandemic have reported that most students were very satisfied with the blended learning mode for improving their learning effectiveness, learning efficiency, and personal learning pace [27]. However, disadvantages of blended learning, such as limited social contact, have also been disclosed [27]. The blended mode used in examinations during the COVID-19 pandemic has also been acknowledged by students for assessment feasibility, test anxiety alleviation, and academic performance improvement [28]. Under the context of the Healthy China Strategy proposed by the National Congress of the Communist Party of China, progress in the construction and application of blended learning in medical education has substantially contributed to the theoretical and practical development of this learning mode [29]. Although the self-reported learning experiences of students might be subjective, they serve as important feedback on the learning mode [1,30]. It is worth noting that for the COVID-19 and post–COVID-19 periods, differences in learning environments might alter students’ needs for the efficiency, availability, and quality of knowledge delivery, which would provide information for the reform of the learning mode under an unexpected public health emergency [22].

However, students’ perceptions of the blended learning mode for undergraduate dental education in both the COVID-19 and post–COVID-19 periods have not been revealed. This study aimed to explore students’ satisfaction and suggestions regarding the blended learning mode in final-year undergraduate dental education during both the COVID-19 and post–COVID-19 periods. Using a questionnaire survey, we gathered students’ satisfaction with blended learning among final-year undergraduate dental students in the West China School of Stomatology of Sichuan University, assessed students’ self-reported desire to engage in blended learning activities, and identified modifiable factors that might influence their potential engagement. Our study reported the learning modes of dental education in the top-ranking dental university during the COVID-19 and post–COVID-19 period.

## Methods

### Study Settings and Participant Criteria

This survey study was undertaken at the West China School of Stomatology of Sichuan University. This school is recognized as a pioneer of modern dental education in China and has consistently ranked first in stomatology evaluations in mainland China [31]. The dental science program at the West China School of Stomatology is the top-ranked discipline among all dental colleges and universities, according to the Ministry of Education of mainland China. The undergraduate dental science (5-year) program is intended to nurture students with basic medicine and clinical medicine knowledge, train students with basic theories and practical skills of stomatology, and qualify students with solid expertise on the diagnosis, treatment, and prevention of common and frequently occurring oral diseases. The course structures and learning modes in this school are considered to represent the highest level of undergraduate dental education in China.

The survey recruited final-year undergraduate dental students who majored in dental science at the West China School of Stomatology of Sichuan University during the end of the fall semester of academic years 2020-2021 and 2023-2024, as representative time points for COVID-19 (December 2019-May 2023) and post–COVID-19 (May 2023), respectively. In total, 248 and 226 final-year undergraduate dental students enrolled in the fall semesters of the academic year 2020 to 2021 and the academic year 2023 to 2024 were invited to participate in the survey. The inclusion criteria were students who (1) were enrolled in the undergraduate stomatology (5 years) program at the West China School of Stomatology of Sichuan University; (2) had completed all previous comprehensive theoretical and practical training courses in the past 4 years; and (3) had smartphone, tablet, and laptop devices with internet access during the data collection process. The exclusion criteria were students who (1) were transfers and (2) were on long-term leave or had dropped out during the study periods (COVID-19 period: December 2019 to May 2023; post–COVID-19 period: May 2023 onward).

### Students’ Exposure to Blended Learning

Participants accessed their blended learning experiences during their final-year study in the fall semester of the academic year 2020 to 2021 and the academic year 2023 to 2024. Courses in the undergraduate stomatology (5 years) program, which is one of China’s double first-class disciplines and is jointly run with the West China Hospital of Stomatology of Sichuan University, were evaluated in this study. This program aims to cultivate talents who have basic medical theoretical and clinical knowledge; master oral medicine theories and clinical operation skills; are engaged in the national medical practitioner qualification certification; and have good professionalism in oral clinical diagnosis, treatment, and prevention. The curricula that were evaluated in this survey consisted of 49 courses divided into 4 types, namely, general courses (10/49, 20%), specialized foundation courses (22/49, 45%), specialized courses (14/49, 29%), and practical courses (3/49, 6%).

### Survey Design and Pilot Test

This study used a self-administered, anonymous, electronic survey, including a survey invitation letter, participant informed consent, a questionnaire of 20 questions, and a free-response item. The first section of the questionnaire collected information on students’ majors and year of study, their self-perceived knowledge levels for blended learning, and the frequency with which they reported using blended learning in their studies. The second section evaluated the types of curricula using blended learning and the detailed implementation of the learning processes. In the third section, the items measured students’ satisfaction with blended learning and factors influencing their satisfaction [32,33]. The following section assessed students’ perceptions of the strengths of blended learning compared with online or offline learning and evaluated their desire to engage in blended learning. The final section asked about factors that might increase or decrease students’ potential engagement [9]. The free-response item was included to gather rich insights into students’ thinking about blended learning. Items assessing students’ self-perceived knowledge levels of blended learning were rated using a 4-point Likert scale (ranging from not knowledgeable to very knowledgeable) [32]. Items assessing students’ satisfaction with blended learning were rated using a 5-point Likert scale (ranging from very dissatisfied to very satisfied) [34]. Items assessing students’ desire to participate in blended learning were rated using desire as a predictor of engagement. Items assessing factors influencing students’ desire to engage in blended learning were evaluated using multiple-choice question options.

The survey was developed on a web-based survey platform, WJX.cn, in mainland China, based on a skip-logic design. The items were developed by a panel of 12 experienced educators, student representatives, and academic affairs experts and were piloted on 10 subjects for 2 rounds without iterative revision. The online survey was conducted following the CHERRIES (Checklist for Reporting Results of Internet E-Surveys) guidelines.

### Data Collection

The survey recruitment was conducted during 2 separate 2-week recruitment periods (January 18 to January 31, 2021, and December 27, 2023, to January 10, 2024). The survey link was included in an invitation letter and was distributed to students via QQ, which is an online platform well accepted by undergraduate students for its instant messaging and online organizing functions [35]. The response settings were rigidly specified so that a respondent could submit only 1 response, and up to 3 reminders were sent to nonresponders during the recruitment period.

### Statistical Analysis

All statistical analyses were performed using SPSS software (version 22.0). The response rates included only fully completed surveys from respondents. Descriptive statistics for each outcome variable were reported as mean, mean difference (MD), and standard deviation (SD) for continuous quantitative variables and frequencies (or percentages) for categorical variables. We conducted the Shapiro-Wilk normality test and found that the student satisfaction data approximately followed a normal distribution across outcome variables (overall blended learning, individual curricula, and specific learning activities). Then, the 1-tailed *t* test was adopted as a parametric test to examine differences between groups by comparing the overall -satisfaction group, the high satisfaction group (HSG, defined as satisfaction responses as very satisfied and satisfied), and the low satisfaction group (LSG, defined as satisfaction responses as very dissatisfied, dissatisfied, and neutral) with a 95% CI. Pearson correlation analysis was used to examine factors associated with higher satisfaction from respondents. The chi-square test, a common nonparametric test for data not assumed to be normally distributed, was used to compare the strengths of blended learning with offline or online learning only between different groups.

### Ethical Considerations

The survey was approved by the Academic Affairs Division, West China School of Stomatology, Sichuan University Institutional Review Board (WCHSIRB-D-2024-481). The electronic survey invitation letter included a description of the study’s objectives and procedures, informed participants that participation was voluntary and anonymous, and clarified that the survey results would be used for research purposes only. Informed consent was obtained from participants upon survey submission. No compensation was given for participating in this study.

## Results

Among the 474 final year undergraduate students recruited in 2020 and 2023, 38% (178/474) completed the survey, with response rates of 46% (114/248) in 2020 and 28% (64/226) in 2023. Of these respondents, 75% (85/114) in 2020 and 73% (47/64) in 2023 described themselves as very knowledgeable or knowledgeable about blended learning. Considering that one response might choose multiple course types, we also analyzed the knowledge levels of responses in 2020 and 2023 (Table 1).

**Table 1:** Characteristics and knowledge levels of the survey in 2020 and 2023.

Curricula		Overall sample^a^, n	Respondents, n (%)			
			Very knowledgeable	Knowledgeable	Somewhat knowledgeable	Not knowledgeable
**2020**						
	General courses	38	3 (8)	22 (58)	13 (34)	0
	Specialized foundation courses	53	8 (15)	33 (62)	12 (23)	0
	Specialized courses	94	13 (14)	63 (67)	17 (18)	1 (1)
	Practical courses	26	5 (19)	19 (73)	1 (4)	1 (4)
**2023**						
	General courses	42 (66)	9 (21)	23 (55)	10 (24)	0
	Specialized foundation courses	38 (59)	5 (13)	23 (61)	10 (26)	0
	Specialized courses	50 (78)	6 (12)	28 (56)	16 (32)	0
	Practical courses	32 (50)	5 (16)	19 (59)	8 (25)	0

^a^The overall sample sizes of specific courses were counted as the sum of all respondents who chose this course.

As for the implementation of blended learning, students reported shifts in the online and offline distribution patterns for specific learning procedures (Multimedia Appendix 1). In general, preclass activities, student sign-in, and in-class quizzes showed less online integration in 2023 compared to 2020. By contrast, teacher-student interactions, student-student interactions, and teachers’ feedback did not follow this pattern, as these procedures showed a decrease in online engagement and an increase in offline engagement in 2023 compared with 2020, which differed from most other teaching procedures. Moreover, practical teaching and final examinations shifted from online to offline and showed almost the same trend in both 2020 and 2023.

Table 2 summarizes students’ satisfaction with blended learning in 2020 and 2023. Overall, students’ satisfaction decreased from 2020 to 2023, as 82% (93/114) versus 59% (38/64) of the students were in the HSG in 2020 versus 2023, and statistically significant differences were found between the HSG and the LSG in both years (MD 1.634 and 95% CI 1.502-1.767 in 2020 and MD 2.116 and 95% CI 2.054-2.177 in 2023). Specifically, compared to 2020, students in 2023 were most satisfied with specialized foundation courses (40/53, 75% in the HSG) and moderately satisfied with general courses (26/38, 68% in the HSG) and specialized courses (63/94, 67% in the HSG), whereas they felt most dissatisfied with practical courses (9/26, 35% in the HSG), and statistically significant differences were found for the mean scores of all curricula (*P*<.001).

**Table 2:** Students’ satisfaction with blended learning for different curriculum types and specific learning activities in 2020 (n=114) and 2023 (n=64).

Terms		Overall (2020 vs 2023)				
		Overall, mean (SD)	HSG^a^, mean (SD)	LSG^b^, mean (SD)	Mean difference (95% CI)	*P* value
Overall curricula		3.79 (0.926) vs 2.36 (1.155)	4.29 (0.457) vs 3.98 (0.513)	2.66 (0.684) vs 1.87 (0.776)	1.634 (1.502-1.767) vs 2.116 (2.054-2.177)	<.001 vs <.001
General courses		3.68 (0.933) vs 2.55 (1.951)	4.19 (0.402) vs 4.27 (0.450)	2.58 (0.793) vs 1.03 (1.425)	1.609 (1.268-1.950) vs 3.238 (3.567-2.909)	<.001 vs <.001
Specialized foundation courses		3.87 (0.900) vs 4.31 (1.720)	4.28 (0.452) vs 4.23 (0.429)	2.62 (0.768) vs 1.12 (1.452)	1.660 (1.341-1.979) vs 3.108 (3.370-2.846)	<.001 vs <.001
Specialized courses		3.75 (0.950) vs 2.92 (1.721)	4.29 (0.455) vs 4.25 (0.440)	2.65 (0.709) vs 1.59 (1.478)	1.641 (1.495-1.786) vs 2.656 (3.030-2.282)	<.001 vs <.001
Practical courses		3.50 (1.105) vs 1.80 (1.904)	4.18 (0.393) vs 4.16 (0.375)	2.22 (0.833) vs 0.80 (1.307)	1.954 (1.516-2.392) vs 3.358 (3.570-3.145)	<.001 vs <.001
Preclass activities		3.79 (0.814) vs 3.75 (0.667)	4.30 (0.462) vs 4.17 (0.377)	2.88 (0.400) vs 2.96 (0.213)	1.423 (1.405-1.442) vs 1.212 (1.189-1.235)	<.001 vs <.001
**Teaching activities**						
	Class content	3.92 (0.754) vs 3.77 (0.636)	4.31 (0.463) vs 4.14 (0.347)	2.94 (0.354) vs 2.95 (0.224)	1.367 (1.342-1.393) vs 1.186 (1.186-1.187)	<.001 vs <.001
	Teaching strategies	3.82 (0.815) vs 3.77 (0.611)	4.31 (0.464) vs 4.14 (0.351)	2.87 (0.409) vs 3.00 (0.000)	1.435 (1.409-1.461) vs 1.140 (1.032-1.247)	<.001 vs <.001
	Examination design	3.82 (0.748) vs 3.60 (0.750)	4.31 (0.464) vs 4.17 (0.378)	2.98 (0.154) vs 2.86 (0.356)	1.330 (1.269-1.391) vs 1.310 (1.320-1.300)	<.001 vs <.001
	Postclass feedback	3.83 (0.740) vs 3.69 (0.639)	4.26 (0.441) vs 4.13 (0.335)	2.95 (0.329) vs 2.96 (0.204)	1.314 (1.304-1.324) vs 1.167 (1.146-1.187)	<.001 vs <.001
	Student engagement	3.71 (0.849) vs 3.77 (0.661)	4.29 (0.459) vs 4.13 (0.344)	2.85 (0.470) vs 2.90 (0.315)	1.446 (1.418-1.475) vs 1.238 (1.287-1.190)	<.001 vs <.001
	Teacher-student interactions	3.75 (0.818) vs 3.64 (0.721)	4.31 (0.465) vs 4.16 (0.370)	2.91 (0.412) vs 2.89 (0.326)	1.396 (1.385-1.405) vs 1.273 (1.283-1.263)	<.001 vs <.001
	Integration between online and offline learning	3.78 (0.750) vs 3.70 (0.728)	4.24 (0.432) vs 4.17 (0.381)	2.93 (0.350) vs 2.87 (0.344)	1.318 (1.306-1.330) vs 1.301 (1.329-1.273)	<.001 vs <.001

^a^HSG: high satisfaction group.

^b^LSG: low satisfaction group.

Regarding students’ attitudes toward individual learning activities in blended learning (Table 2), statistically significant differences were found in the overall mean satisfaction scores for all learning activity items (*P*<.001). In addition, statistically significant differences (*P*<.001) were found between the HSG and the LSG in the mean scores for all learning activity items in both 2020 and 2023. Students expressed the highest satisfaction for class content, with an overall mean satisfaction score of 3.92 (SD 0.754) in 2020 versus 3.77 (SD 0.636) in 2023. In addition, students were more satisfied with student engagement during blended learning in 2023 (mean 3.71, SD 0.849) compared to 2020 (mean 3.77, SD 0.661). By contrast, students expressed lower satisfaction with learning activities such as preclass activities, teaching strategies, examination design, postclass feedback, teacher-student interactions, and integrations between online and offline learning in 2023 compared to 2020.

Factors correlated with students’ satisfaction with blended learning were analyzed using Pearson correlation analysis (Multimedia Appendix 2). Among different online learning materials, MOOC (*r*=0.19; *P*=.04) was significantly correlated with students’ satisfaction in 2020, while live lectures (*r*=0.28; *P*=.03) and other materials (*r*=0.29; *P*=.02) were significantly correlated with students’ satisfaction in 2023. Regarding offline learning materials, designated learning materials (*r*=0.20; *P*=.03) were significantly associated with higher satisfaction with blended learning in 2020. Other materials, such as clinical cases, literature materials, students’ self-chosen materials, and clinical teaching models, were not related to student satisfaction in 2020. The 2023 results were similar to those of 2020, with most offline learning materials showing no correlation with students’ satisfaction, except for other materials (*r*=0.29; *P*=.02). In addition, among all the teaching procedures, teachers’ feedback was significantly correlated with higher student satisfaction (*r*=0.32; *P*=.01) in 2023. Other face-to-face instructions, including collaborative learning, student-student interactions, teacher-student interactions, in-class quizzes, and so on, had no significant correlation with students’ satisfaction in 2020 and 2023. Moreover, there were also significant associations between students’ satisfaction and active learning strategies, such as turn and talk (*r*=0.39; *P*=.001), pause procedures during lecture (*r*=0.35; *P*=.004), and other strategies (*r*=0.29; *P*=.02) in 2023, while only simulation exercises were significantly correlated with higher student satisfaction in 2020 (*r*=0.19; *P*=.04).

As shown in Table 3, students perceived the strengths of blended learning compared to online learning or offline learning only. Compared to traditional offline learning in 2020 versus 2023, students valued blended learning for its ability to enhance learning motivation (*P*<.001), communication skills (*P*=.008), practical skills (*P*<.001), course interest (*P*=.04), time and space flexibility (*P*<.001), and new media literacy skills (*P*=.002). When compared to online learning in 2020 versus 2023, students appreciated blended learning for improving learning motivation (*P*=.04) and new media literacy skills (*P*=.02).

**Table 3:** Students’ self-perceived strengths of blended learning compared to offline learning or online learning only in 2020 (N=114) versus 2023 (N=64).

Terms	Blended vs offline (2020 vs 2023)		Blended vs online (2020 vs 2023)		*P* value (2020 vs 2023)
	n (%)	95% CI	n (%)	95% CI	
Knowledge acquisition	63 (55) vs 30 (47)	46.1-64.1 vs 34.3-59.4	61 (54) vs 37 (58)	44.4-62.4 vs 45.4-70.3	.40 vs .22
Learning motivation	84 (74) vs 45 (70)	64.9-80.9 vs 58.8-81.8	54 (47) vs 29 (45)	38.4-56.5 vs 32.8-57.9	<.001 vs .04
Communication skills	44 (39) vs 34 (53)	30.2-47.8 vs 40.6-65.7	62 (54) vs 27 (42)	45.3-63.2 vs 29.8-54.6	.008 vs .22
Collaboration skills	53 (47) vs 35 (55)	37.6-55.6 vs 42.2-67.2	43 (38) vs 29 (45)	29.4-46.9 vs 32.8-57.9	.09 vs .29
Practical skills	17 (15) vs 27 (42)	9.5-22.6 vs 29.8-54.6	54 (47) vs 34 (53)	9.5-22.6 vs 40.6-65.7	<.001 vs .22
Interest in courses	25 (22) vs 19 (30)	15.3-30.4 vs 18.2-41.2	37 (33) vs 20 (31)	25.6-41.5 vs 19.6-42.9	.04 vs .85
Time and space flexibility	82 (72) vs 35 (55)	63.1-79.4 vs 42.2-67.2	48 (42) vs 24 (38)	33.5-51.3 vs 25.3-49.7	<.001 vs .05
New media literacy skills	54 (47) vs 32 (50)	38.4-56.5 vs 37.4-62.6	33 (29) vs 19 (30)	21.4-37.9 vs 18.2-41.2	.002 vs .02
Expanded horizons and mindset	30 (26) vs 20 (31)	19.1-35.1 vs 19.6-42.9	23 (20) vs 14 (22)	13.8-28.5 vs 11.5-32.3	.14 vs .23
None of the above	11 (10) vs 8 (13)	5.5-16.5 vs 4.2-20.8	8 (7) vs 5 (8)	3.6-13.2 vs 1.1-14.6	.24 vs .38

Most students expressed a preference for incorporating blended learning in final-year undergraduate dental education, with 70% (80/114) of the students in 2020 and 61% (39/64) in 2023 indicating a desire to engage in blended learning. Multimedia Appendix 3 summarizes that the leading factors decreasing students’ potential engagement in blended learning were the same in both 2020 and 2023, namely, unstable technical support (68/114, 60% in 2020 vs 26/64, 41% in 2023), poor integration between online and offline learning (58/114, 51% in 2020 vs 34/64, 53% in 2023), a lack of learning motivation (51/114, 45% in 2020 vs 24/64, 38% in 2023), and a lack of teacher-student interaction (44/114, 39% in 2020 vs 20/64, 31% in 2023). Compared to 2020, there was a decline in unstable technical support, a lack of learning motivation, a lack of teacher-student interaction, and a lack of teaching materials, which decreased students’ desire to engage in blended learning. By contrast, the influences of improper performance assessment, insufficient teaching ability, and limited teaching supervision and evaluation on decreasing students’ engagement were upregulated in 2023.

On the other hand, the top factors increasing students’ potential engagement in blended learning were the same in both 2020 and 2023, namely, providing high-quality learning materials (76/114, 67% in 2020 vs 43/64, 67% in 2023) and the improvement of the technical environment (62/114, 54% in 2020 vs 35/64, 55% in 2023). Compared to 2020, the influence of the use of teacher incentives (59/114, 52% in 2020 vs 22/64, 34% in 2023) on increasing students’ engagement decreased in 2023. By contrast, the impacts of emphasizing evaluation in the learning process (51/114, 45% in 2020 vs 33/64, 52% in 2023) and establishing effective classroom schedules (48/114, 42% in 2020 vs 31/64, 48% in 2023) on enhancing students’ engagement increased in 2023.

For the free-response items, students suggested ways to enhance engagement in blended learning, including involving students in curriculum design and learning material development, more emphasis on problem solving during face-to-face instruction, as well as offering incentives during the learning process, etc.

## Discussion

### Principal Findings

This study demonstrates that final-year undergraduate dental students had a generally satisfied attitude toward blended learning in the COVID-19 and post–COVID-19 years. Students acknowledged the strengths of blended learning in multiple aspects compared to online or offline learning only. Meanwhile, students desired to engage in blended learning and reported factors that might influence their potential engagement. These findings reveal students’ self-reported satisfaction and perceptions regarding blended learning for undergraduate dental education and provide practical clues for enhancing student engagement in blended learning in the circumstance of the COVID-19 pandemic and beyond.

At its peak, the COVID-19 pandemic significantly impacted higher education by necessitating rapid shifts to online or blended learning in health professional training [7,9,36]. Participants generally acknowledged that the diverse methodologies in online-based learning during the COVID-19 pandemic advanced the modernization of health professional education [37]. In addition, comparable or even greater effectiveness for knowledge acquisition than nonblended learning has been observed in health professionals, although heterogeneity should be noted [38]. Similar positive effects of blended learning have been reported in health professions students for learning effectiveness [39], graduate medical students for knowledge gain [40], and undergraduate medicine and nursing students for knowledge outcomes and clinical skills training [7,41]. Serving as one of the most prevalent teaching approaches for medical education during the pandemic, blended learning encountered challenges such as students’ limited acceptance of technology-assisted learning, difficulties in student engagement during the learning process, and issues related to cognition and retention [42]. Moreover, the sudden transition of medical education to online-only or blended learning modes during the pandemic might interrupt academic interactions and trigger unstructured learning patterns among health professional students and arouse their need for retaining certain course components, such as practical training in traditional face-to-face learning [43].

In our survey study, more students showed a generally satisfied attitude toward blended learning in 2020 compared to 2023 (Table 2). Other studies have shown that students’ age, computer experience, and technology acceptance might affect their satisfaction with online learning [44,45], while failure to incorporate these aspects in our survey might bias the results. In fact, final-year dental students complete most of their theoretical and practical studies during their junior year or earlier, making them familiar with online learning environments and technology for blended learning, which helps minimize bias in our study. Notably, students expressed the lowest degree of satisfaction toward practical courses in both 2020 and 2023 (Table 2), consistent with the finding that the number of students who desired to engage in practical courses involving blended learning was the least among all curriculum types in both years (data not shown). However, in another study assessing student perceptions of blended learning in practical training in orthodontic education, students expressed general acceptance and a preference for engagement in the blended learning mode compared with both traditional solid models and e-models [18]. Specifically, the blended learning mode that combines electrical 3D simulation models with traditional plaster models cultivates critical thinking and haptic sensitivity to facilitate clinical practice [18]. Another retrospective study concluded that the integration of a flipped classroom approach into blended learning in a dental anatomy course could effectively improve students’ scores in practical assessments, although not in theoretical assessments [46]. These studies support the adoption of blended learning in practical training for undergraduate dental education. Moreover, the learning of experiential forms of knowledge is also important for dental skills [47,48]. By contrast, in the COVID-19 pandemic context, the emphasis on patients’ knowledge of dental emergencies, disease transmission, and needed preparations underscored the need for dental students to enhance their professional preparedness and knowledge for clinical practice [38]. This might fuel senior dental students’ aspiration to acquire clinical skills, particularly during the post–COVID-19 period. Future instruction on the rationality of course design, the availability of reliable online resources, and the delivery of in-class activities is necessary for the implementation of blended learning in dental courses with practical components, improving learner acceptance and performance outcomes.

In our study, students showed the lowest satisfaction for teacher-student interactions in both 2020 and 2023 (Table 2), and teacher-student interactions were identified as a key factor influencing student engagement in blended learning (Multimedia Appendix 3). According to the social constructivist theory [49], interactions between a student and their teachers and peers during blended learning could stimulate effective learning and thus improve student learning [50]. Blended learning can also integrate the advantages of synchronous teacher-student interactions with asynchronous time and space flexibility to foster the engagement of students and teachers in the learning process [51,52]. Indeed, teacher-student interactions contribute to improved student engagement and satisfaction with blended learning compared to online learning and face-to-face learning [50]. During the COVID-19 pandemic, students reported insufficient interactions with teachers for preclinical practice in dental education, and such reduced teacher-student interactions partially impacted students’ overall satisfaction with online learning-involved processes [9,50,53]. Barriers such as challenges in selecting helpful peers, limited and delayed teacher feedback and support, as well as difficulty in discerning relevant information, particularly in asynchronous online environments, might lead to students’ dissatisfaction with teacher-student interaction experiences in blended learning.

Students greatly appreciated the strengths of blended learning over online or offline learning only (Table 3). In the past decade, online learning resources and platforms have substantially facilitated the successful implementation of blended learning in higher education [54]. During the COVID-19 pandemic, more high-quality online courses were freely developed, opened, and shared among different schools to enhance public accessibility for educators and students [55]. In recent years, it is encouraging that more high-quality online learning platforms with communication components have been available for web-based courses in dental education, such as Rain Classroom [56] and PMPHMOOC [57]. These platforms could be accessed by students and teachers on campus or remotely via mobile devices, such as laptops, tablets, and phones [9]. Moreover, social software tools, such as Tencent QQ chat groups [9] and WeChat apps [58], also demonstrate an educational function by promoting multidirectional interactions, including teacher-student interactions, even during the COVID-19 pandemic [11]. Although these platforms are mostly limited to China and might not be applicable to other countries, the fast-growing evolution of these online learning platforms and social tools is progressing toward meeting the needs of dental students and educators in the COVID-19 and post–COVID-19 periods.

Students expressed a desire to engage in blended learning in both 2020 and 2023 and reported several factors that might influence their potential engagement. More than half of the students indicated the importance of tech for blended learning in both years (Multimedia Appendix 3). As mentioned earlier, much effort is needed to facilitate the implementation of higher education due to the COVID-19 pandemic [59]. However, the availability of newly developed educational resources is still limited. Moreover, their applications and adaptations to a wider range require more time and practice [35,60]. Previous studies have shown a strong relationship between the learning environment and student learning behaviors, self-perceived satisfaction, and academic performance [61-64]. As a well-established element highly susceptible to the environment, self-regulated learning imposes higher standards for students to adapt to the inevitably varied COVID-19 pandemic and postpandemic world than before [19,36]. Efforts have been made to cultivate self-regulated skills in students for a web-based environment, such as the provision of tutorials before starting a course or the supply of real-time skill guidance within a course. Our findings also suggest that with the incorporation of blended learning, a significant number of students viewed the impact of the development of the learning environment positively when faced with the unprecedented COVID-19 and post–COVID-19 periods.

### Limitations

There are several limitations of this study. First, the survey was performed in a single dental school and might not reflect the blended learning experiences of other schools. Second, our survey did not record students’ health status during the COVID-19 and post-COVID-19 periods.

### Conclusions

This survey study demonstrates that final-year undergraduate dental students are generally satisfied with blended learning in both the COVID-19 and post–COVID-19 years. Although students prefer to engage in blended learning compared with online or offline learning only, their desire to participate might vary depending on multiple factors. Dental schools, policy makers, and educators could increase students’ engagement in blended learning by incorporating more teacher-student interactions, establishing standardized assessment mechanisms, providing dedicated learning materials and platforms, and promoting self-regulated learning among dental students. The COVID-19 pandemic has raised an alarm for dental education to reform into a more flexible and adaptable mode, and this survey provides clues for developing a new face of blended learning–involved dental education in the context of the pandemic and beyond.

## Data Availability

The data that support the findings of this study are available from the corresponding author upon reasonable request.

## Appendix

Multimedia Appendix 1 Characteristics of online vs offline distribution patterns of specific teaching procedures in blended learning in 2020 vs 2023.

Multimedia Appendix 2 Characteristics of online vs offline distribution patterns of specific teaching procedures in blended learning in 2020 vs 2023.

Multimedia Appendix 3 Factors influencing final-year undergraduate dental students’ potential engagement in blended learning during and after COVID-19 in 2020 and 2023.

## Abbreviations

CHERRIES: Checklist for Reporting Results of Internet E-Surveys
HSG: high satisfaction group
LSG: low satisfaction group
MD: mean difference

## References

[1] Chen Y, Deng J, Li B, Yang Y, He Z, Ye L, Zhang L, Ren Q, Zheng Q (2022). Curriculum setting and students' feedback of pre-clinical training in different dental schools in China-a national-wide survey. Eur J Dent Educ.

[2] Wang YH, Zhao Q, Tan Z (2017). Current differences in dental education between Chinese and Western models. Eur J Dent Educ.

[3] Manogue M, McLoughlin J, Christersson C, Delap E, Lindh C, Schoonheim-Klein M, Plasschaert A (2011). Curriculum structure, content, learning and assessment in European undergraduate dental education - update 2010. Eur J Dent Educ.

[4] Gatt G, Attard NJ (2023). Multimodal teaching methods for students in dentistry: a replacement for traditional teaching or a valuable addition? A three-year prospective cohort study. BMC Med Educ.

[5] Du GF, Li CZ, Shang SH, Xu XY, Chen HZ, Zhou G (2013). Practising case-based learning in oral medicine for dental students in China. Eur J Dent Educ.

[6] Huang B, Zheng L, Li C, Li L, Yu H (2013). Effectiveness of problem-based learning in Chinese dental education: a meta-analysis. J Dent Educ.

[7] Li L, Liu X, Chen Z, Wang L, Lian X, Zou H (2021). The application of a case-based social media-assisted teaching method in cariology education: comparative study. J Med Internet Res.

[8] Santos GN, Leite AF, Figueiredo PT, Pimentel NM, Flores-Mir C, de Melo NS, Guerra EN, De Luca Canto G (2016). Effectiveness of e-learning in oral radiology education: a systematic review. J Dent Educ.

[9] Wang K, Zhang L, Ye L (2021). A nationwide survey of online teaching strategies in dental education in China. J Dent Educ.

[10] Mangione S, Nieman LZ, Greenspon LW, Margulies H (1991). A comparison of computer-assisted instruction and small-group teaching of cardiac auscultation to medical students. Med Educ.

[11] Rouse DP (2000). The effectiveness of computer-assisted instruction in teaching nursing students about congenital heart disease. Comput Nurs.

[12] Tomej K, Liburd J, Blichfeldt BS, Hjalager AM (2022). Blended and (not so) splendid teaching and learning: higher education insights from university teachers during the Covid-19 pandemic. Int J Educ Res Open.

[13] Vallée A, Blacher J, Cariou A, Sorbets E (2020). Blended learning compared to traditional learning in medical education: systematic review and meta-analysis. J Med Internet Res.

[14] Bonnel WB, Starling CK, Wambach KA, Tarnow K (2003). Blended roles: preparing the advanced practice nurse educator/clinician with a web-based nurse educator certificate program. J Prof Nurs.

[15] Chen M, Ye L, Weng Y (2022). Blended teaching of medical ethics during COVID-19: practice and reflection. BMC Med Educ.

[16] Cheng X, Mo W, Duan Y (2023). Factors contributing to learning satisfaction with blended learning teaching mode among higher education students in China. Front Psychol.

[17] Reissmann DR, Sierwald I, Berger F, Heydecke G (2015). A model of blended learning in a preclinical course in prosthetic dentistry. J Dent Educ.

[18] Ho AC, Liao C, Lu J, Shan Z, Gu M, Bridges SM, Yang Y (2022). 3-Dimensional simulations and student learning in orthodontic education. Eur J Dent Educ.

[19] Kavadella A, Tsiklakis K, Vougiouklakis G, Lionarakis A (2012). Evaluation of a blended learning course for teaching oral radiology to undergraduate dental students. Eur J Dent Educ.

[20] (2020). WHO Director-General's opening remarks at the media briefing on COVID-19 - 11 March 2020. World Health Organization.

[21] Coronavirus disease (COVID-19) pandemic. World Health Organization.

[22] Chilton JK, Hanks S, Watson HR (2024). A blended future? A cross-sectional study demonstrating the impacts of the COVID-19 pandemic on student experiences of well-being, teaching and learning. Eur J Dent Educ.

[23] Choi B, Jegatheeswaran L, Minocha A, Alhilani M, Nakhoul M, Mutengesa E (2020). The impact of the COVID-19 pandemic on final year medical students in the United Kingdom: a national survey. BMC Med Educ.

[24] Long N, Wolpaw DR, Boothe D, Caldwell C, Dillon P, Gottshall L, Koetter P, Pooshpas P, Wolpaw T, Gonzalo JD (2020). Contributions of health professions students to health system needs during the COVID-19 pandemic: potential strategies and process for U.S. medical schools. Acad Med.

[25] Pellerone M (2021). Self-perceived instructional competence, self-efficacy and burnout during the Covid-19 pandemic: a study of a group of Italian school teachers. Eur J Investig Health Psychol Educ.

[26] Garzon Diaz K, Villamil Duarte A, Velasco Forero S, Herrera Jacobo S (2024). Post-pandemic face-to-face learning environments for undergraduate students: a scoping review protocol. PLoS One.

[27] Nijakowski K, Lehmann A, Zdrojewski J, Nowak M, Surdacka A (2021). The effectiveness of the blended learning in conservative dentistry with endodontics on the basis of the survey among 4th-year students during the COVID-19 pandemic. Int J Environ Res Public Health.

[28] Hong S, Go B, Rho J, An S, Lim C, Seo DG, Ihm J (2023). Effects of a blended design of closed-book and open-book examinations on dental students' anxiety and performance. BMC Med Educ.

[29] Ma J, Jiang X, Wang J, Liang Z, Sun Z, Qian H, Gong A (2022). The construction and application of a blended teaching model under the strategic background of healthy China. Biochem Mol Biol Educ.

[30] Davey J, Bryant ST, Dummer PM (2015). The confidence of undergraduate dental students when performing root canal treatment and their perception of the quality of endodontic education. Eur J Dent Educ.

[31] Global ranking of academic subjects. ShanghaiRanking.

[32] Bucklin BA, Asdigian NL, Hawkins JL, Klein U (2021). Making it stick: use of active learning strategies in continuing medical education. BMC Med Educ.

[33] AlRuthia Y, Alhawas S, Alodaibi F, Almutairi L, Algasem R, Alrabiah HK, Sales I, Alsobayel H, Ghawaa Y (2019). The use of active learning strategies in healthcare colleges in the Middle East. BMC Med Educ.

[34] Ren L, Ren J, Liu C, He M, Qiu X (2022). Policy goals of contract arrangements in primary care in jeopardy: a cross-sectional consumer satisfaction survey of community residents in Hangzhou, China. Front Public Health.

[35] Zhao Y, Watterston J (2021). The changes we need: education post COVID-19. J Educ Chang.

[36] Lee M, An SY, Ihm J (2024). Dental students' satisfaction with web-based learning during the initial phase of the COVID-19 pandemic: mixed methods study. J Med Internet Res.

[37] Iyer PH (2024). Corona virus disease (COVID-19): lessons learned impact on the education of health professionals. Adv Exp Med Biol.

[38] Liu Q, Peng W, Zhang F, Hu R, Li Y, Yan W (2016). The effectiveness of blended learning in health professions: systematic review and meta-analysis. J Med Internet Res.

[39] Cook DA, Levinson AJ, Garside S, Dupras DM, Erwin PJ, Montori VM (2008). Internet-based learning in the health professions: a meta-analysis. JAMA.

[40] Jwayyed S, Stiffler KA, Wilber ST, Southern A, Weigand J, Bare R, Gerson LW (2011). Technology-assisted education in graduate medical education: a review of the literature. Int J Emerg Med.

[41] Enoch LC, Abraham RM, Singaram VS (2023). Factors that enhance and hinder the retention and transfer of online pre-clinical skills training to facilitate blended learning. Adv Med Educ Pract.

[42] Kaup S, Jain R, Shivalli S, Pandey S, Kaup S (2020). Sustaining academics during COVID-19 pandemic: the role of online teaching-learning. Indian J Ophthalmol.

[43] O'Brien L, Tighe J, Doroud N, Barradell S, Dowling L, Pranata A, Ganderton C, Lovell R, Hughes R (2023). "Burnout felt inevitable": experiences of university staff in educating the nursing and allied health workforce during the first COVID-19 waves. Front Public Health.

[44] McCutcheon K, Lohan M, Traynor M, Martin D (2015). A systematic review evaluating the impact of online or blended learning vs. face-to-face learning of clinical skills in undergraduate nurse education. J Adv Nurs.

[45] Moule P, Ward R, Lockyer L (2010). Nursing and healthcare students' experiences and use of e-learning in higher education. J Adv Nurs.

[46] Bakr MM, Massey WL, Massa HM (2016). Flipping a dental anatomy course: a retrospective study over four years. Educ Res Int.

[47] Whipp JL, Ferguson DJ, Wells LM, Iacopino AM (2000). Rethinking knowledge and pedagogy in dental education. J Dent Educ.

[48] Vanka A, Vanka S, Wali O (2020). Flipped classroom in dental education: a scoping review. Eur J Dent Educ.

[49] Westerlaken M, Christiaans-Dingelhoff I, Filius RM, de Vries B, de Bruijne M, van Dam M (2019). Blended learning for postgraduates; an interactive experience. BMC Med Educ.

[50] Ullah R, Siddiqui F, Adnan S, Afzal AS, Sohail Zafar M (2021). Assessment of blended learning for teaching dental anatomy to dentistry students. J Dent Educ.

[51] Blond N, Chaux AG, Hascoët E, Lesclous P, Cloitre A (2024). Blended learning compared to traditional learning for the acquisition of competencies in oral surgery by dental students: a randomized controlled trial. Eur J Dent Educ.

[52] Sellnow-Richmond D, Strawser MG, Sellnow DD (2019). Student perceptions of teaching effectiveness and learning achievement: a comparative examination of online and hybrid course delivery format. Commun Teach.

[53] Di Giacomo P, Di Paolo C (2021). COVID-19 and dental distance-based education: students' perceptions in an Italian university. BMC Med Educ.

[54] Schlenz MA, Schmidt A, Wöstmann B, Krämer N, Schulz-Weidner N (2020). Students' and lecturers' perspective on the implementation of online learning in dental education due to SARS-CoV-2 (COVID-19): a cross-sectional study. BMC Med Educ.

[55] Quinn B, Field J, Gorter R, Akota I, Manzanares MC, Paganelli C, Davies J, Dixon J, Gabor G, Amaral Mendes R, Hahn P, Vital S, O'Brien J, Murphy D, Tubert-Jeannin S (2020). COVID-19: the immediate response of European academic dental institutions and future implications for dental education. Eur J Dent Educ.

[56] Rain classroom web version. Rain Classroom.

[57] People's health MOOC homepage. People's Health MOOC.

[58] Luan H, Wang M, Sokol RL, Wu S, Victor BG, Perron BE (2020). A scoping review of WeChat to facilitate professional healthcare education in Mainland China. Med Educ Online.

[59] Wang C, Cheng Z, Yue XG, McAleer M (2020). Risk management of COVID-19 by universities in China. J Risk Financial Manag.

[60] Hattar S, AlHadidi A, Sawair FA, Alraheam IA, El-Ma'aita A, Wahab FK (2021). Impact of COVID-19 pandemic on dental education: online experience and practice expectations among dental students at the University of Jordan. BMC Med Educ.

[61] Divaris K, Barlow PJ, Chendea SA, Cheong WS, Dounis A, Dragan IF, Hamlin J, Hosseinzadeh L, Kuin D, Mitrirattanakul S, Mo’nes M, Molnar N, Perryer G, Pickup J, Raval N, Shanahan D, Songpaisan Y, Taneva E, Yaghoub‐Zadeh S, West K, Vrazic D (2008). The academic environment: the students’ perspective. Eur J Dent Educ.

[62] DiMattio MJ, Hudacek SS (2020). Educating generation Z: psychosocial dimensions of the clinical learning environment that predict student satisfaction. Nurse Educ Pract.

[63] Han F, Ellis RA (2023). The relations between self-reported perceptions of learning environment, observational learning strategies, and academic outcome. J Comput High Educ.

[64] Wang H, Li D, Gu C, Wei W, Chen J (2023). Research on high school students' behavior in art course within a virtual learning environment based on SVVR. Front Psychol.

