# Supplementary material for: Dental Undergraduate Students’ Perceptions of Blended Learning in the COVID-19 and Post–COVID-19 Years: Survey Study
Source: JMIR Form Res. 2025 Nov 28;9:e63453. doi: 10.2196/63453 (PMC12701352; doi:10.2196/63453)
Supplement: Multimedia Appendix 1 [file formative_v9i1e63453_app1.docx]

**Multimedia Appendix 1.** Characteristics of online vs offline distribution patterns of specific teaching procedures in blended learning in 2020 vs 2023.

| Teaching procedure | Online vs Offline, 2020 | Online vs Offline, 2023 |
| --- | --- | --- |
| Pre-class activities | 74% vs 24% 50 | 66% vs 30% 36 |
| Student sign-in | 77% vs 21% 56 | 59% vs 39% 20 |
| Teacher-student interactions | 57% vs 42% 15 | 41% vs 38% 3 |
| Student-student interactions | 61% vs 36% 25 | 42% vs 53% 11 |
| Collaborative learning | 42% vs 52% 10 | 15% vs 79% 64 |
| In-class quiz | 75% vs 23% 52 | 67% vs 31% 36 |
| Teachers^,^ feedback | 54% vs 43% 11 | 38% vs 57% 19 |
| Practical teaching | 11% vs 86% 75 | 14% vs 84% 70 |
| Final exams | 11% vs 86% 75 | 10% vs 88% 78 |
