# Supplementary material for: Dental Undergraduate Students’ Perceptions of Blended Learning in the COVID-19 and Post–COVID-19 Years: Survey Study
Source: JMIR Form Res. 2025 Nov 28;9:e63453. doi: 10.2196/63453 (PMC12701352; doi:10.2196/63453)
Supplement: Multimedia Appendix 2 [file formative_v9i1e63453_app2.docx]

**Multimedia Appendix 2.** Factors correlated with students’ satisfaction for blended learning in dental education for 2020 vs 2023.

| Subgroup | Pearson correlation analysis, 2020 vs 2023 | | |
| --- | --- | --- | --- |
|  | r | 95% CI | P value |
| **Online learning materials** | | | |
| Pre-recorded video or audio | 0.06 vs 0.11 | -0.12-0.25 vs -0.14-0.35 | .50 vs .38 |
| Live lecture | 0.04 vs 0.28 | -0.15-0.22 vs 0.04-0.49 | .70 vs .03 |
| Student self-chosen materials | 0.05 vs -0.07 | -0.14-0.23 vs -0.31-0.18 | .63 vs .57 |
| MOOC | 0.19 vs 0.14 | 0.01-0.36 vs -0.11-0.37 | .04 vs .28 |
| Designated online resources | -0.14 vs 0.08 | -0.31-0.05 vs -0.17-0.32 | .15 vs .54 |
| Others | -0.02 vs 0.29 | -0.21-0.16 vs 0.05-0.50 | .82 vs .02 |
| **Offline learning materials** | | | |
| Literature materials | 0.14 vs 0.06 | -0.05-0.31 vs -0.19-0.30 | .15 vs .66 |
| Clinical cases | 0.05 vs 0.01 | -0.14-0.23 vs -0.24-0.25 | .60 vs .97 |
| Designated learning materials | 0.20 vs 0.09 | 0.02-0.37 vs -0.16-0.33 | .03 vs .48 |
| Students’ self-chosen materials | 0.08 vs 0.03 | -0.11-0.26 vs -0.21-0.28 | .42 vs .80 |
| Clinical teaching models | -0.01 vs 0.01 | -0.20-0.17 vs -0.24-0.25 | .90 vs .97 |
| Others | 0.08 vs 0.29 | -0.10-0.26 vs 0.05-0.50 | .39 vs .02 |
| **Teaching procedures** | | | |
| Teachers’ feedback | -0.02 vs 0.32 | -0.21-0.16 vs 0.09-0.53 | .81 vs .01 |
| Collaborative learning | 0.05 vs 0.11 | -0.14-0.23 vs -0.14-0.35 | .61 vs .39 |
| Student-student interactions | 0.17 vs 0.19 | -0.01-0.34 vs -0.06-0.41 | .07 vs .14 |
| Teacher-student interactions | 0.05 vs 0.19 | -0.13-0.23 vs -0.06-0.42 | .61 vs .14 |
| In-class quiz | 0.08 vs -0.07 | -0.11-0.26 vs -0.31-0.18 | .41 vs .60 |
| Pre-class activities | 0.09 vs 0.05 | -0.10-0.27 vs -0.20-0.29 | .36 vs .69 |
| Student sign-in | 0.05 vs 0.09 | -0.13-0.23 vs -0.16-0.32 | .58 vs .50 |
| Practical teaching | 0.10 vs 0.13 | -0.09-0.28 vs -0.12-0.36 | .29 vs .32 |
| Final exams | 0.13 vs 0.24 | -0.06-0.30 vs -0.00-0.46 | .18 vs .05 |
| **Use of active learning strategy** | | | |
| Collaborative learning | 0.09 vs 0.13 | -0.10-0.27 vs -0.12-0.37 | .34 vs .30 |
| Hands-on game | 0.18 vs 0.02 | -0.00-0.36 vs -0.22-0.27 | .05 vs .86 |
| Flipped classroom | -0.05 vs 0.24 | -0.23-0.13 vs -0.01-0.46 | .58 vs .06 |
| Turn and Talk | 0.08 vs 0.39 | -0.11-0.26 vs 0.16-0.58 | .40 vs .001 |
| Student presentation | 0.03 vs 0.09 | -0.16-0.21 vs -0.16-0.33 | .79 vs .50 |
| Case-based discussion | 0.12 vs -0.14 | -0.07-0.30 vs -0.37-0.11 | .21 vs .28 |
| Small group discussion | 0.11 vs -0.03 | -0.08-0.29 vs -0.28-0.21 | .25 vs .79 |
| One-minute paper | 0.03 vs 0.20 | -0.16-0.21 vs -0.05-0.43 | .79 vs .11 |
| Pause procedures during lecture | 0.13 vs 0.35 | -0.05-0.31 vs 0.12-0.55 | .16 vs .004 |
| Bulleted breaks during lecture | 0.06 vs 0.17 | -0.13-0.24 vs -0.08-0.40 | .55 vs .19 |
| Simulation exercises | 0.19 vs 0.04 | 0.01-0.37 vs -0.21-0.28 | .04 vs .76 |
| Others | 0.02 vs 0.29 | -0.17-0.20 vs 0.05-0.50 | .84 vs .02 |
