# Supplementary figures and images for: Dental Undergraduate Students’ Perceptions of Blended Learning in the COVID-19 and Post–COVID-19 Years: Survey Study
Source: JMIR Form Res. 2025 Nov 28;9:e63453. doi: 10.2196/63453 (PMC12701352; doi:10.2196/63453)

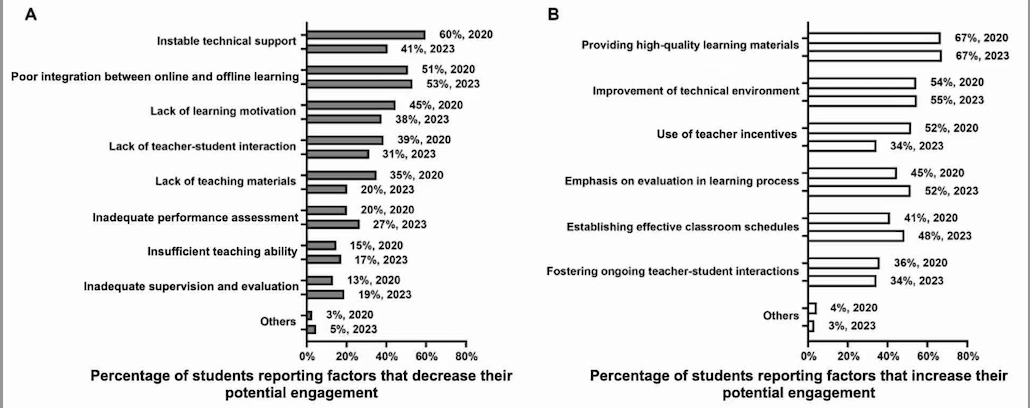

Supplement: Multimedia Appendix 3 [file formative_v9i1e63453_app3.png]
